# Elevated Levels of NLRP3 in Cerebrospinal Fluid of Patients With Autoimmune GFAP Astrocytopathy

**DOI:** 10.3389/fneur.2019.01019

**Published:** 2019-10-17

**Authors:** Ying Luo, Wei Yan, Zheyi Zhou, Baozhu Liu, Zhanhang Wang, Jinyu Chen, Honghao Wang

**Affiliations:** ^1^Department of Neurology, Nanfang Hospital, Southern Medical University, Guangzhou, China; ^2^Department of Neurology, The First People's Hospital of Kashgar Prefecture, Kashgar, China; ^3^Department of Neurology, Liuzhou Traditional Chinese Medical Hospital, The Third Affiliated Hospital of Guangxi University of Chinese Medicine, Liuzhou, China; ^4^Department of Neurology, Guangdong 999 Brain Hospital, Guangzhou, China

**Keywords:** autoimmune GFAP astrocytopathy, neuroinflammation, EDSS score, NLRP3 inflammasome, inflammatory cytokines

## Abstract

Autoimmune glial fibrillary acidic protein (GFAP) astrocytopathy, a newly defined autoimmune encephalitis, is an antibody-mediated meningoencephalomyelitis. The pathogenesis of the disease is still unclear. Nod-like receptor protein 3 (NLRP3) inflammasome is a complex composed of a variety of proteins that recognizes a variety of ligands and ultimately leads to the development of inflammatory responses. This is important for infectious, inflammatory, and immune diseases. The aims of this study were to detect levels of cerebrospinal fluid (CSF) NLRP3 inflammasome and inflammatory factors in autoimmune GFAP astrocytopathy patients and to study the relationships between these profiles. Twenty autoimmune GFAP astrocytopathy patients, 17 viral meningoencephalitis (VM) patients, and 16 controls (CTLs) were recruited. The levels of NLRP3 inflammasomes, interleukin (IL)-1β, IL-6, and IL-17 were measured by enzyme-linked immunosorbent assay (ELISA). The Expanded Disability Status Scale (EDSS) score was used to assess the severity of clinical manifestations. The results showed that the levels of NLRP3 inflammasome and inflammatory cytokines (IL-1β, IL-6, IL-17) were significantly more elevated in CSF of patients with autoimmune GFAP astrocytopathy than that in CTLs. When compared with VM patients, significantly elevated NLRP3 inflammasome was found in GFAP astrocytopathy patients, while the levels of IL-1β, IL-6, and IL-17 were not different between the two groups. Significant positive correlations were found between NLRP3 inflammasome and inflammatory cytokines and they were all positively related to the severity of the disease. Moreover, we found that patients with positive anti-GFAP antibodies had higher levels of NLRP3 and inflammatory factors. And the severity of the disease was positively correlated with GFAP antibody titers. Taken together, the results suggested that NLRP3 inflammasome was involved in the pathogenesis of autoimmune GFAP astrocytopathy. It can be used to assess the severity of the disease or act as a new target for the therapy.

## Introduction

As an important component of mature astrocyte intermediate filaments, GFAP plays prominent roles in maintaining the morphology of astrocytes, providing the blood-brain barrier (BBB) integrity and regulating synaptic function (1). Autoimmune GFAP astrocytopathy, defined by Fang et al. (2), is an antibody-mediated meningoencephalomyelitis. Its pathogenesis remains unclear. Though short-term responsiveness to immunotherapy may be achieved, it is characterized by frequent recurrence. GFAP antibody in serum or CSF is considered to be its biomarker (2). The main symptoms and signs include subacute headache, myelopathic symptoms, optic disc edema, tremor, ataxia, neck stiffness, autonomic instability, confusion, and progressive cognitive impairment (3–5), which were considerably similar to the symptoms of other autoimmune encephalitis. Lesions include the subcortical white matter, hypothalamus, brainstem, basal ganglia, cerebellum, and spinal cord. The characteristic MRI feature is brain linear perivascular radial gadolinium enhancement in white matter perpendicular to ventricle (6). However, there is no accurate and uniform diagnostic criteria for Autoimmune GFAP astrocytopathy at present, and the detection of other coexisting neural autoantibodies in the same patient makes diagnosis difficult.

The inflammasome expressed mainly in monocytes and macrophages (7) is a complex composed of a variety of proteins that regulate the activation of caspases-1 and further promote the maturation and secretion of cytokine precursors pro-IL-1β and pro-IL-18 during natural immune defense (8, 9). There are four main types of inflammasome that have been discovered, namely NLRP1 inflammasome, NLRP3 inflammasome, NLRC4 inflammasome, and AIM2 inflammasome (10). NLRP3 is the most extensive studied one. Researchers have reported that NLRP3 inflammasome is associated with a range of aseptic inflammatory diseases such as diabetes (11), arteriosclerosis (12), systemic lupus erythematosus (13), rheumatoid arthritis (14), and central nervous diseases such as Alzheimer's disease (AD) and Parkinson's disease (PD) (15, 16). Our previous studies also found that the levels of NLRP3 were significantly elevated in anti-N-methyl- D-aspartate receptor (NMDAR) encephalitis, neuromyelitis optica (NMO) and multiple sclerosis (MS). At the same time, the data analysis implied that the concentrations of NLRP3 were related to the severity and prognosis of these diseases (related articles are being submitted). However, whether the concentration of NLRP3 is related to autoimmune GFAP astrocytopathy is still unclear.

Therefore, we detected levels of CSF NLRP3 inflammasome and inflammatory factors in autoimmune GFAP astrocytopathy patients and studied the relationships between levels of CSF NLRP3 and inflammatory factors, or EDSS score, or concentrations of anti-GFAP antibodies in this study.

## Materials and Methods

### Patients and Controls

A total of 20 autoimmune GFAP astrocytopathy patients, 17 age- and gender- matched viral meningoencephalitis (VM) patients and 16 CTLs with non-inflammatory neurological diseases were prospectively enrolled from the Department of Neurology, Nanfang Hospital of Southern Medical University. The inclusion criteria for the autoimmune GFAP astrocytopathy patient group was based on the diagnosis criteria published by Boyan Fang in 2016, mainly for typical clinical manifestations and serum or CSF anti-GFAP antibody positive (2). In this study, 13 patients were anti-GFAP antibodies positive in both serum and CSF, while 7 patients were only seropositive. CSF and serum antibodies were negative in both VM and CTL groups. Clinical features of all patients and controls are shown in Table 1. All patients were treated at Nangfang Hospital of Southern Medical University. This study was approved by the Ethics Committee of the Nanfang Hospital of Southern Medical University, and written informed consent was obtained from each participant.

**Table 1:** The clinical characteristics of autoimmune GFAP astrocytopathy, VM patients, and controls.

	**Autoimmune GFAP Astrocytopathy (*n =* 20)**	**VM (*n =* 17)**	**CTLs (*n =* 16)**
Gender (Male/Female)	13/7	9/8	11/5
Age (years)	38.70 ± 11.50	33.59 ± 13.94	33.90 ± 2.00
**Psychiatric and neurologic symptoms**			
Fever	16 (80%)	12 (71%)	–
Disorders of	7 (35%)	7 (41%)	–
memory, behavior,			
and cognition			
Seizures	4 (20%)	4 (24%)	–
Autonomic	5 (25%)	0 (0%)	–
disturbances			
Disturbance of	5 (25%)	0 (0%)	–
consciousness			
Abnormal	13 (65%)	4 (24%)	–
movements			
ovarian teratoma	1 (5%)	0 (0%)	–
**Lesion location**			
Brain	16 (80%)	–	–
Spinal cord	2 (10%)	–	–
Brian and spinal	2 (10%)	–	–
cord			
CSF NLPR3	4.18 ± 2.42	1.72 ± 0.90	0.92 ± 0.45
(ng/ml, mean ± SD)			
CSF IL-1β (pg/ml,	0.90 ± 1.15	0.74 ± 1.26	0.08 ± 0.08
mean ± SD)			
CSF IL-6 (pg/ml,	6.98 ± 2.79	6.21 ± 5.58	2.53 ± 0.43
mean ± SD)			
CSF IL-17A	6.84 ± 5.16	4.07 ± 1.49	2.64 ± 2.15
(pg/ml, mean ± SD)			
EDSS scores	3 (1, 5.5)	–	–
[median (minimum-			
maximum)]			
**Anti-GFAP antibody**			
SE (positive/total)	20/20	0/17	0/16
CSF (positive/total)	13/20	0/17	0/16

*CSF, Cerebrospinal Fluid; SE, serum; VM, viral meningoencephalitis patients; CTLs, controls*.

### Clinic Evaluation

All autoimmune GFAP astrocytopathy patients were hospitalized during the onset. The EDSS score was applied to evaluate disease severity. The definition of clinical relapse was the same as before, a sudden appearance of new symptoms, lasting for at least 24 h, with an increase in EDSS scores of >1.0 before sampling. All samples were collected before the patients received immunomodulating therapies.

### Preparation of CSF Samples

Lumbar puncture was performed to collect CSF for diagnostic purposes and further CSF analysis. For anti-GFAP antibody-positive CSF, further dilutions were performed to determine antibody titer. After diagnosis, the CSF samples were aliquoted into polypropylene tubes and stored at −80°C until the assays were performed.

### Enzyme-Linked Immunosorbent Assay (ELISA)

Commercially available ELISA kits were used to quantify the concentrations of NLRP3 inflammasome (CSB-E15885h, Cusabio, Wuhan, china) in CSF according to the manufacturers' instructions. The minimum detectable dose of NLRP3 was 0.039 ng/mL. Computer software and regression analysis were used to create standard curve, and the fit of the optimal standard curve is *r*^2^ = 0.99. ELISA kits were also used to detect levels of CSF IL-1β, IL-6, and IL-17 (Bender MedSystems GmbH, Vienna, Austria).

### Statistical Analysis

Data were presented as mean ± standard deviation or median (minimum-maximum). Independent samples between different groups were compared using non-parametric tests. Correlations between the profiles were assessed using Spearman's rank analysis or Pearson's correlation analysis, as appropriate. All statistical analyses were performed using Statistical Package for the Social Sciences (SPSS) software, release 20.0 (IBM Corp., Armonk, NY, USA). A value of *P* < 0.05 was considered statistically significant.

## Results

### The Clinical Features of the Patients With Anti-GFAP Antibody

We collected clinical manifestations of the patients with anti-GFAP antibody in Nanfang hospital. Among the 20 patients, some clinical manifestations were prominent, such as fever (*n* = 16, 80%), abnormal movements (*n* = 13, 65%) and disorders of memory, behavior and cognition (*n* = 7, 35%). Other symptoms include seizures (*n* = 4, 20%), autonomic disturbances (*n* = 5, 25%), disturbance of consciousness (*n* = 5, 25%), and ovarian teratoma (*n* = 1, 5%). From the MRI information, we found that lesions were involved in the brain (*n* = 16,80%), spinal cord (*n* = 4,20%), and brain-spinal cord (*n* = 2,10%). The specific positions included white matter, meninges, basal ganglia, ventricle, and spinal cord. The clinical data was shown in Table 1.

### NLRP3 Inflammasome and Inflammatory Cytokines of CSF in Different Groups

**Figure 1:**
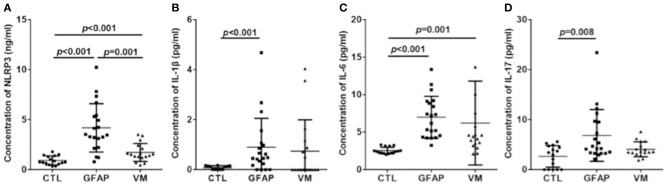
CSF levels of NLRP3 and inflammatory cytokines in different groups. CSF levels of NLRP3 and inflammatory cytokines were determined by ELISA in autoimmune GFAP astrocytopathy patients (*n* = 20), VM patients (*n* = 17) and controls (*n* = 16). Changes of CSF NLRP3 **(A)**, IL-1β **(B)**, IL-6 **(C)**, and IL-17 **(D)** are shown. Results are mean ± SD from all samples performed in duplicate. The non-parametric test was used for statistical analysis and a *p*-value of 0.05 or less was considered as significant (GFAP, autoimmune; GFAP, astrocytopathy).

The levels of CSF NLRP3 inflammasome, IL-1β, IL-6, and IL-17 were detected in patients and control groups using ELISA. Results were shown in Figure 1. Mean NLRP3 (ng/ml) was 4.18 ± 2.42 for GFAP astrocytopathy, compared to 1.72 ± 0.90 for VM and 0.92 ± 0.45 for CTLs. Concentrations of CSF NLRP3 inflammasome in the GFAP astrocytopathy group were significantly higher than those in VM (*p* = 0.001, Figure 1A) and CTLs (*p* < 0.001, Figure 1A). At the same time, the levels of NLRP3 inflammasome in the VM group were also significantly higher than that in CTLs (*p* < 0.001, Figure 1A). Levels of CSF IL-1β, IL-6, and IL-17 of GFAP astrocytopathy patients were significantly higher than those of controls (*p* < 0.001, *p* < 0.001, *p* = 0.008, respectively, Figures 1B–D). However, they were not different between the GFAP astrocytopathy patients and VM patients (Figures 1B–D).

### Relationships Between CSF NLRP3 Inflammasome and CSF Cytokines

**Figure 2:**
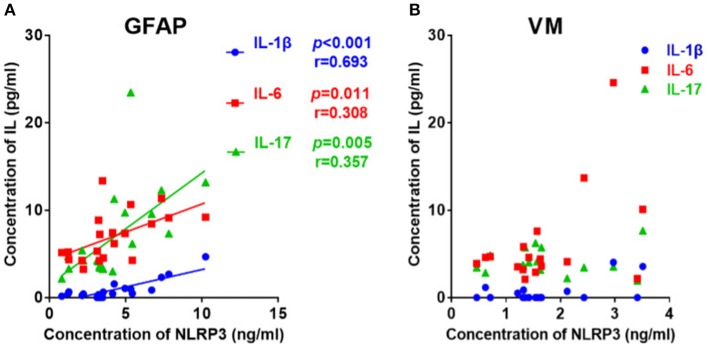
The correlations between the CSF levels of NLRP3 and inflammatory cytokines in patients with autoimmune GFAP astrocytopathy or VM. **(A)** The correlations between the CSF levels of NLRP3 and inflammatory cytokines in patients with autoimmune GFAP astrocytopathy are shown. **(B)** The correlations between the CSF levels of NLRP3 and inflammatory cytokines in patients with VM are shown. Pearson's correlation analysis was used for statistical analysis and a *p*-value of 0.05 or less was considered as significant.

Considering that both NLRP3 inflammasome and cytokines concentrations were elevated in the GFAP astrocytopathy group, we examined the correlations between them. The NLRP3 inflammasome was positively correlated with IL-1β (*r* = 0.693, *p* < 0.001), IL-6 (*r* = 0.308, *p* = 0.011), and IL-17 (*r* = 0.357, *p* = 0.005) in CSF (Figure 2). At the same time, we examined the correlations between NLRP3 inflammasome and cytokines in VM group. However, the NLRP3 had nothing to do with the cytokines in VM group (Figure 2B).

### Relationships Between CSF Profiles and Clinical Severity

**Figure 3:**
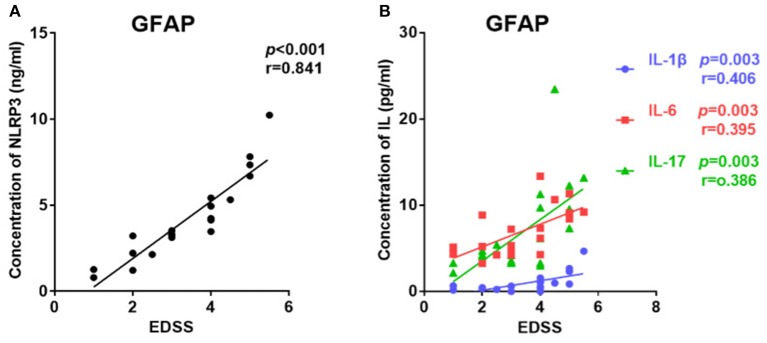
The correlations between the EDSS scores and CSF levels of NLRP3, IL-1β, IL-6, and IL-17 in patients with autoimmune GFAP astrocytopathy. **(A)** The correlations between the EDSS scores and CSF levels of NLRP3 in autoimmune GFAP astrocytopathy patients are shown. **(B)** The correlations between the EDSS scores and CSF levels of IL-1β, IL-6, and IL-17 in patients with autoimmune GFAP astrocytopathy are shown. Spearman's rank analysis was used for statistical analysis and a *p*-value of 0.05 or less was considered as significant.

The EDSS score was used to evaluate disease severity. The median EDSS score was 3 (1–5.5) for the GFAP astrocytopathy group. In the GFAP astrocytopathy group, patients with higher NLRP3 concentrations showed severe disease disability, and there was a significant positive correlation between them (*r* = 0.841, *p* < 0.001, Figure 3A). Similarly, IL-1β, IL-6, and IL-17 were also significantly associated with EDSS (IL-1β: *r* = 0.406, *p* = 0.003; IL-6: *r* = 0.395, *p* = 0.003; IL-17: *r* = 0.386, *p* = 0.003) in the GFAP astrocytopathy group (Figure 3B).

### CSF NLRP3 Inflammasome and Inflammatory Cytokines in Subgroups of Autoimmune GFAP Astrocytopathy

**Figure 4:**
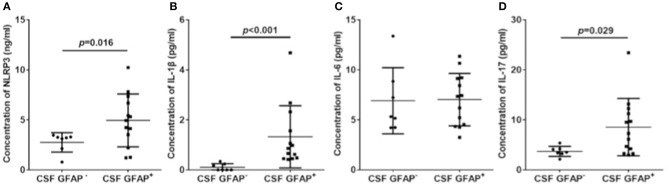
CSF NLRP3 inflammasome and inflammatory cytokines in subgroups of autoimmune GFAP astrocytopathy. Autoimmune GFAP astrocytopathy patients were divided into two subgroups (GFAP^+^ and GFAP^−^). Changes of CSF NLRP3 **(A)**, IL-1β **(B)**, IL-6 **(C)**, and IL-17 **(D)** in different subgroups are shown. Results are the mean ± SD from all samples performed in duplicate. The non-parametric test was used for statistical analysis and a *p*-value of 0.05 or less was considered as significant.

Among the patients with GFAP astrocytopathy, 13 of 20 were anti-GFAP antibody positive in the CSF. Therefore, we divided GFAP astrocytopathy cases into two subgroups (CSF GFAP^+^ and CSF GFAP^−^) (clinical features are shown in Table 2) and compared levels of CSF NLRP3 inflammasome and inflammatory cytokines in different subgroups. The results showed that patients with positive anti-GFAP antibody in CSF had higher NLRP3 inflammasome (*p* = 0.016, Figure 4A), IL-1β (*p* < 0.001, Figure 4B), and IL-17 (*p* = 0.029, Figure 4D), while there was no difference in IL-6 between the two subgroups (Figure 4C).

**Table 2:** The clinical characteristics of subgroups of autoimmune GFAP astrocytopathy.

	**GFAP^**+**^ (*n =* 13)**	**GFAP^**−**^ (*n =* 7)**
Gender (Male/Female)	10/3	3/4
Age (years)	35.77 ± 11.50	43.00 ± 14.00
**Psychiatric and neurologic symptoms**		
Fever	11 (85%)	5 (71%)
Disorders of memory, behavior, and	5 (38%)	2 (29%)
cognition		
Seizures	3 (23%)	1 (14%)
Autonomic disturbances	5 (38%)	0 (0%)
Disturbance of consciousness	4 (31%)	1 (14%)
Abnormal movements	10 (77%)	3 (43%)
Ovarian teratoma	1 (8%)	0 (0%)
CSF NLPR3 (ng/ml, mean ± SD)	4.95 ± 2.63	2.75 ± 0.97
CSF IL-1β (pg/ml, mean ± SD)	1.32 ± 1.24	0.11 ± 0.14
CSF IL-6 (pg/ml, mean ± SD)	7.02 ± 2.62	6.91 ± 3.31
CSF IL-17A (pg/ml, mean ± SD)	8.54 ± 5.73	3.70 ± 1.00
EDSS scores [median (minimum-	4 (1, 5.5)	3 (1, 4)
maximum)]		
**Anti-GFAP antibody**		
SE	Positive	Positive
CSF	Positive	Negative

### Relationships Between CSF Profiles and Anti-GFAP Antibody Titers

**Figure 5:**
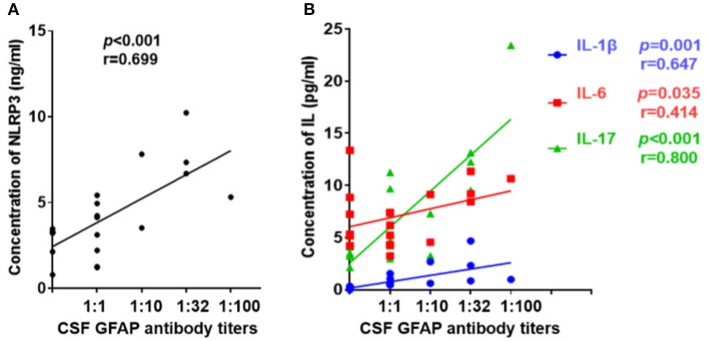
Relationship between CSF profiles and anti-GFAP antibody titer. Anti-GFAP antibody titer was detected and the relationships between CSF profiles and anti-GFAP antibody titers were analyzed. **(A)** Relationship between CSF NLRP3 and anti-GFAP antibody titers. **(B)** Relationships between CSF IL-1β, IL-6, and IL-17 and anti-GFAP antibody titers. Spearman's rank analysis was used for statistical analysis, and a *p*-value of 0.05 or less was considered as significant.

Further, we diluted the CSF at 1:10, 1:32, and 1:100 to detect anti-GFAP antibody titers and analyzed the relationships between CSF profiles and anti-GFAP antibody titers. Results indicated that the anti-GFAP antibody titers had significant positive correlations with the levels of NLRP3 inflammasome (*r* = 0.699, *p* < 0.001, Figure 5A), IL-1β (*r* = 0.647, *p* = 0.001), IL-6 (*r* = 0.414, *p* = 0.035), and IL-17 (*r* = 0.800, *p* < 0.001) (Figure 5B).

### Relationship Between EDSS Scores and Anti-GFAP Antibody Titers

**Figure 6:**
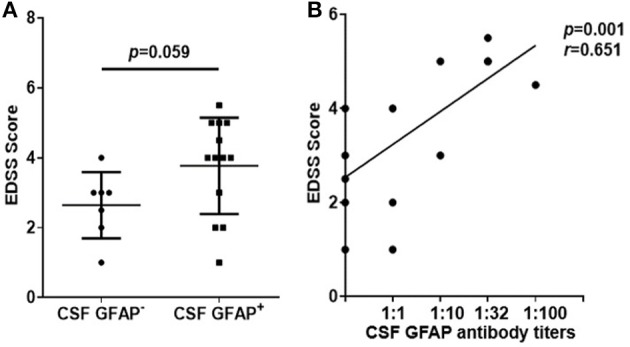
Relationship between EDSS scores and anti-GFAP antibody titers. **(A)** Changes of the EDSS scores of two subgroups of autoimmune GFAP astrocytopathy. The non-parametric test was used for statistical analysis. **(B)** The association of antibody titers with EDSS scores. Spearman's rank analysis was used for statistical analysis.

Similarly, we studied the effect of anti-GFAP antibody level on disease severity. First, we compared the EDSS scores of two subgroups of autoimmune GFAP astrocytopathy. We found that the GFAP^+^ group had a higher EDSS score than the GFAP^−^ group, but there was no statistical difference (*p* = 0.059, Figure 6A). Then, we analyzed the association of antibody titers with EDSS scores. A significant positive correlation was found between them (*r* = 0.651, *p* = 0.001, Figure 6B).

## Discussion

In the present study, we found elevated levels of NLRP3 inflammasome and inflammatory cytokines (IL-1β, IL-6, IL-17) in CSF in patients with autoimmune GFAP astrocytopathy. There were significant positive correlations between NLRP3 and each cytokine, and they were all positively related to the severity of the disease. Moreover, we found that patients with positive anti-GFAP antibodies had higher levels of NLRP3 and inflammatory factors. And the severity of the disease was positively correlated with GFAP antibody titers. We hypothesized that these conditions might be associated with the process of pyroptosis mediated by NLRP3 inflammasomes.

As a newly defined autoimmune encephalitis, autoimmune GFAP astrocytopathy has no well-defined specific clinical feature and diagnosis, and pathogenesis remains unclear. Some histopathology findings of brain biopsies from patients with autoimmune GFAP astrocytopathy have been reported to gain insight into its pathogenesis. Biopsy revealed a marked inflammatory response in the perivascular area and T and B lymphocytes were present in the lesion (4, 5). Plasma cells, monocytes, and macrophages have also been found to be widely present in the brain tissues of patients with autoimmune GFAP astrocytopathy (17). In the GFAP antibody-related CNS idiopathic inflammatory disease-necrotizing meningoencephalitis and granulomatous meningoencephalitis, the infiltration of inflammatory cells (CD3^+^ T cells, B lymphocytes, plasma cells, and macrophage) of the perivascular region was the most important feature of them (18, 19). Therefore, we believed that the neuroinflammation resulting from the interaction of lymphocytes, microglia, macrophages, and antibodies secreted by plasma cells is the most likely pathogenesis of autoimmune GFAP astrocytopathy.

As a basic innate immune response in the CNS, neuroinflammation plays an important role in the response to pathogen and host-derived cell damage in brain and spinal cord. It removes invading substances, damaged cells, and promotes tissue repair (20–22). Recognition of microbes and damage signals by receptors of the innate immune system is a trigger of neuroinflammation. Toll-like receptors (TLRs) and nucleotide-binding oligomerization domain (NOD)-like receptors (NLRs) are the two most important receptors (23). Upon detection of microbial (pathogen-associated molecular patterns, PAMPs) or cell damage molecular patterns (damage-associated molecular pattern, DAMPs), NLRs bind to the adaptor protein ASC and activate the caspase-1 to initiate the formation of multiprotein intracellular complexes to regulate the secretion of the IL-1 cytokine family (24–26). In the NLRs, NLRP3 is the most fully described inflammasome at present.

NLRP3 can recognize a variety of ligands and provide a platform for caspase-1 activation, leading to the maturation and secretion of cytokine precursors pro-IL-1β and pro-IL-18 and ultimately leading to the development of inflammatory responses, which are important for many diseases such as infectious, inflammatory, and immune diseases (27–30). Activation of caspase-1 induces endogenous death of inflammatory cells, ultimately leading to the release of pro-inflammatory intracellular substances in the cells (31). Recent studies have shown that levels of IL-1β and IL-18 in the brain tissue, plasma and CSF in patients with neurodegenerative diseases, brain injury and CNS infection are elevated, suggesting that IL-1β and IL-18 are involved in neuroinflammation (32–34). Binding of IL-1β and IL-18 to their respective receptors triggers a complex signaling event profile, leading to the expression of multiple inflammation-related genes (35). IL-1β promotes the activation of astrocytes, microglia, and infiltrating T cells in the CNS, then induces the production of pro-inflammatory cytokines such as IL-6 and TNF-α (36). Moreover, IL-1β promotes the differentiation of mouse and human-naive T cells into T-helper 17 (Th17) cells that produce the pro-inflammatory cytokine IL-17 (37, 38). IL-18 can induce the generation of chemokines and pro-inflammatory cytokines in natural killer cells, Th1 cells and B cells to stimulate the immune responses mediated by Th cells (39, 40). In addition, IL-18 can induce the expression of pro-inflammatory cytokines and caspase-1 by activating the signaling pathways in microglia (41).

Overall, in this study, we found significant increases in the levels of NLRP3 inflammasome and its downstream cytokines such as IL-1β, IL-6, and IL-17 in the CSF of patients with autoimmune GFAP astrocytopathy. There was also a significant positive correlation between them. This suggests that the NLRP3 inflammasome is significantly activated in autoimmune GFAP astrocytopathy patients and triggers a downstream inflammatory response that is involved in the pathogenesis of the disease.

Moreover, the CSF levels of NLRP3 inflammasome and its downstream cytokines were positively correlated with DESS scores, further confirming that NLRP3 inflammasome promoted the development of the neuroinflammation. This suggests that NLRP3 inflammasome can be used to assess the severity of autoimmune GFAP astrocytopathy and acts as a new target for the therapy.

In addition, we also found higher levels of NLRP3 and inflammatory factors in patients with anti-GFAP antibodies. And the severity of the disease was positively correlated with GFAP antibody titers. This mechanism is still unclear. We suspect that it may be related to astrocyte damage, and further research is needed.

## Data Availability Statement

All datasets generated for this study are included in the manuscript.

## Ethics Statement

The studies involving human participants were reviewed and approved by Natural Science Foundation of China and Guangdong Provincial Science and Technology plan projects. The patients/participants provided their written informed consent to participate in this study. Written informed consent was obtained from the individual(s) for the publication of any potentially identifiable images or data included in this article.

## Author Contributions

HW and JC designed experiments and revised the article. YL and BL collected patients' CSF samples and clinical data, completed the experiment, and wrote the manuscript. ZZ and WY analyzed data and organized pictures. ZW revised the article.

### Conflict of Interest

The authors declare that the research was conducted in the absence of any commercial or financial relationships that could be construed as a potential conflict of interest.
